# 4,4′-Bipyridinium bis­(2-carboxy­pyridine-3-carboxyl­ate)

**DOI:** 10.1107/S1600536808042220

**Published:** 2008-12-17

**Authors:** Janet Soleimannejad, Hossein Aghabozorg, Ali Morsali, Foruzan Hemmati, Faranak Manteghi

**Affiliations:** a Department of Chemistry, Ilam University, Ilam, Iran; b Faculty of Chemistry, Tarbiat Moallem University, Tehran, Iran; c Department of Chemistry, School of Sciences, Tarbiat Modarres University, PO Box 14155-4838, Tehran, Iran; d Faculty of Chemistry, Payame Noor University (PNU), Abhar, Iran; e Department of Chemistry, Iran University of Science and Technology, Tehran, Iran

## Abstract

The title salt, C_10_H_10_N_2_^2+^·2C_7_H_4_NO_4_^−^ or (4,4′-bpyH_2_)(py-2,3-dcH)_2_, prepared by the reaction between pyridine-2,3-dicarboxylic acid (py-2,3-dcH_2_) and 4,4′-bipyridine (4,4′-bpy), consists of two anions and one centrosymmetric dication. In the crystal, there are two strong O—H⋯O hydrogen bonds involving the two carboxyl­ate groups, with an O⋯O distance of 2.478 (1) Å, and an N—H⋯N hydrogen bond between the anion and cation, with an N⋯N distance of 2.743 (1) Å. These inter­actions, along with other O—H⋯O and C—H⋯O hydrogen bonds, π–π stacking [centroid–centroid distances 3.621 (7) and 3.612 (7) Å] and ion pairing, lead to the formation of the three-dimensional structure.

## Related literature

For proton-transfer ion pairs, see: Seethalakshmi *et al.* (2007 ▶); Manteghi *et al.* (2007 ▶); Aghabozorg, Manteghi & Ghadermazi (2008 ▶). For the use of ion pairs for the formation of metal organic frameworks, see: Aghabozorg, Manteghi & Sheshmani (2008 ▶). For hydrogen bonding, see: Desiraju & Steiner (1999 ▶).
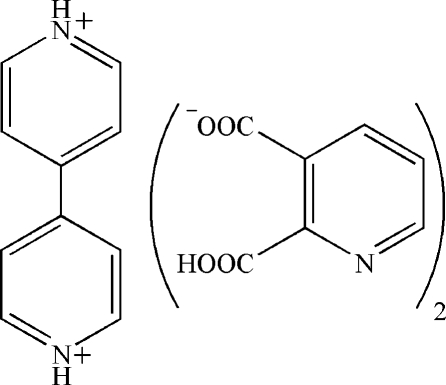


## Experimental

### 

#### Crystal data

C_10_H_10_N_2_^2+^·2C_7_H_4_NO_4_^−^
*M* *_r_* = 490.42
Monoclinic, 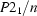

*a* = 6.6675 (2) Å
*b* = 13.7755 (5) Å
*c* = 11.5887 (4) Å
β = 106.310 (2)°
*V* = 1021.56 (6) Å^3^
*Z* = 2
Mo *K*α radiation
μ = 0.12 mm^−1^
*T* = 120 (2) K
0.33 × 0.25 × 0.10 mm

#### Data collection

Bruker SMART CCD area-detector diffractometer
Absorption correction: multi-scan (*SADABS*; Bruker, 2001 ▶) *T*_min_ = 0.904, *T*_max_ = 0.988
18931 measured reflections
2327 independent reflections
2053 reflections with *I* > 2σ(*I*)
*R*_int_ = 0.028

#### Refinement

*R*[*F* ^2^ > 2σ(*F* ^2^)] = 0.036
*wR*(*F* ^2^) = 0.105
*S* = 1.05
2327 reflections
163 parameters
H-atom parameters constrained
Δρ_max_ = 0.31 e Å^−3^
Δρ_min_ = −0.28 e Å^−3^

### 

Data collection: *SMART* (Bruker, 2007 ▶); cell refinement: *SAINT* (Bruker, 2007 ▶); data reduction: *SAINT*; program(s) used to solve structure: *SHELXS97* (Sheldrick, 2008 ▶); program(s) used to refine structure: *SHELXL97* (Sheldrick, 2008 ▶); molecular graphics: *SHELXTL* (Sheldrick, 2008 ▶); software used to prepare material for publication: *SHELXTL*.

## Supplementary Material

Crystal structure: contains datablocks I, global. DOI: 10.1107/S1600536808042220/su2083sup1.cif

Structure factors: contains datablocks I. DOI: 10.1107/S1600536808042220/su2083Isup2.hkl

Additional supplementary materials:  crystallographic information; 3D view; checkCIF report

## Figures and Tables

**Table 1:** Hydrogen-bond geometry (Å, °)

*D*—H⋯*A*	*D*—H	H⋯*A*	*D*⋯*A*	*D*—H⋯*A*
N2—H2*A*⋯N1	0.85	1.90	2.7430 (14)	175
O4—H4*A*⋯O2^i^	0.85	1.64	2.4782 (12)	171
C3—H3⋯O4^ii^	0.95	2.40	3.2055 (15)	143
C4—H4⋯O2^iii^	0.95	2.55	3.4324 (15)	155
C9—H9⋯O1^iv^	0.95	2.41	3.3405 (15)	166
C11—H11⋯O1^v^	0.95	2.52	3.4610 (15)	170
C12—H12⋯O3^v^	0.95	2.19	2.9004 (15)	131

Symmetry codes: (i) 
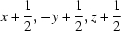
; (ii) 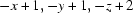; (iii) 
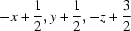
; (iv) 
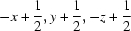
; (v) 
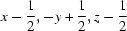
.

**Fig. 1.**
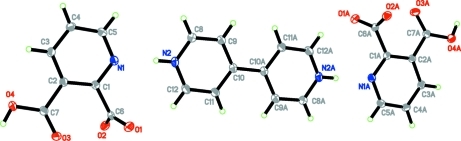
The molecular structure of the title compound showing the displacement ellipsoids drawn at the 50% proability level.

**Fig. 2.**
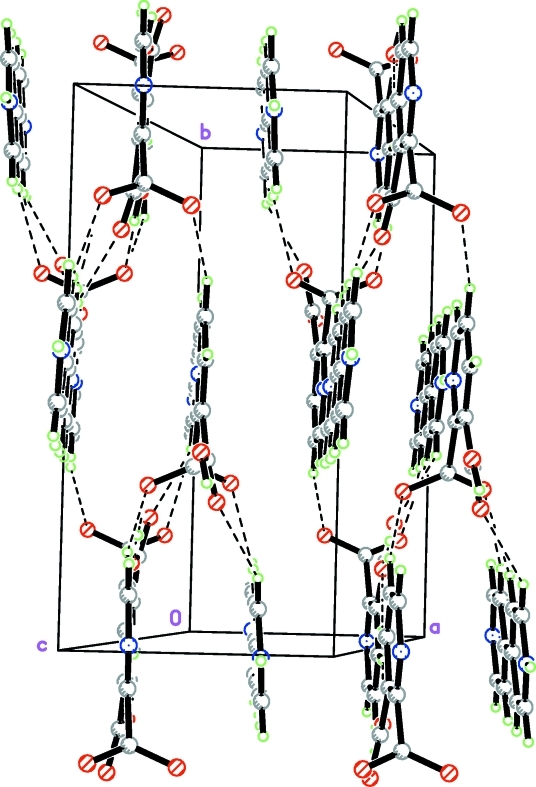
A view of the crystal packing diagram of the title compound with the hydrogen bonds shown as dashed lines.

**Fig. 3.**
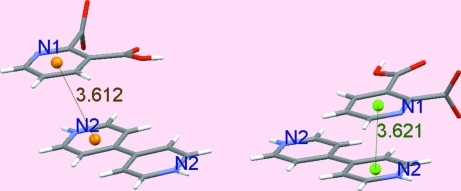
The π-π stacking in the title compound, between acid (N1/C1—C5) and base (N2/C8—C12) rings with symmetry codes: right-hand-side = -x, 1 - y, 1 - z; left-hand-side = 1 - x, 1 - y, 1 - z.

**Fig. 4.**
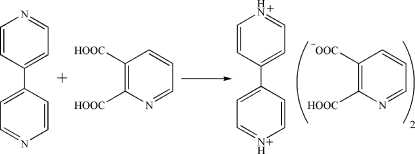
The formation of the title compound.

## supplementary crystallographic information

### Comment

Up to now, pyridine-2,3-dicarboxylic acid has been used to synthesize a numer of proton transfer ion pairs, such as 1,4-Diazoniabicyclo[2.2.2]octane bis(3-carboxypyridine-2-carboxylate) 2.17-hydrate (Seethalakshmi *et al., *2007), propane-1,3-diaminium pyridine-2,3-dicarboxylate monohydrate (Manteghi *et al., *2007) and piperazinediium bis(2-carboxypyridine-3-carboxylate) (Aghabozorg, Manteghi & Ghadermazi, 2008). The last two have been used to synthesize some metal organic frameworks (Aghabozorg, Manteghi, Sheshmani, 2008) in which the acid acts as a mono- or dianionic fragment. In the title ion pair, (4,4'-bpyH_2_)(py-2,3-dcH)_2_, the centrosymmetric dicationic moiety is balanced by two acid moieties in the monoanionic form, as shown in Fig. 1.

In the crystal structure various O—H···O, N—H···N and C—H···O hydrogen bonds are present (Table 1 and Fig. 2). The N2—H2A···N1 hydrogen bond, classified as very strong (Desiraju & Steiner, 1999), links directly the cation and anion of the centrosymmetric unit, with a 5° deviation from linearity and a distance of 2.743 (4) Å.

There is also π-π stacking (Fig. 3) between the acid (N1/C1—C5) and the base (N2/C8—C12) rings with different symmetry codes (-*x*, 1 - *y*, 1 - *z* and 1 - *x*, 1 - *y*, 1 - *z*) at distances of 3.621 (7) and 3.612 (7) Å, respectively. As shown by the torsion angles, C2-C1-C6-O2 and C2-C1-C6-O1 [78.49 (14)° and -105.43 (3)°, respectively], it can be concluded that the carboxylate group, involving atoms O1 and O2, is almost perpendicular to the π-ring of the acid. However, torsion angles, C3-C2-C7-O4 and C3-C2-C7-O3 [15.5 (2)° and -162.6 (1)°, respectively], indicate that the carboxylate groups, involving atoms O3 and O4, are nearly coplanar with the ring.

### Experimental

An aqueous solution (10 ml) of 4,4'-bipyridine (156 mg, 1 mmol) and pyridine-2,3-dicarboxylic acid (167 mg, 1 mmol) was refluxed for two hours. Yellow crystals of the title compound were obtained from the solution after two hours at room temperature.

### Refinement

The H-atoms were included in calculated positions and treated as riding atoms: O-H = 0.85 Å, N-H = 0.85 Å, C-H = 0.95 Å with *U*_iso_(H) =1.2*U*_eq_(parent O, N or C-atom).

### Crystal data

**Table:**

C_10_H_10_N_2_^2+^·2C_7_H_4_NO_4_^−^	*F*(000) = 508
*M**_r_* = 490.42	*D*_x_ = 1.594 Mg m^−^^3^
Monoclinic, *P*2_1_/*n*	Mo *K*α radiation, λ = 0.71073 Å
Hall symbol: -P 2yn	Cell parameters from 7505 reflections
*a* = 6.6675 (2) Å	θ = 2.4–27.5°
*b* = 13.7755 (5) Å	µ = 0.12 mm^−^^1^
*c* = 11.5887 (4) Å	*T* = 120 K
β = 106.310 (2)°	Block, yellow
*V* = 1021.56 (6) Å^3^	0.33 × 0.25 × 0.10 mm
*Z* = 2	

### Data collection

**Table:**

Bruker SMART CCD area-detector diffractometer	2327 independent reflections
Radiation source: fine-focus sealed tube	2053 reflections with *I* > 2σ(*I*)
graphite	*R*_int_ = 0.028
φ and ω scans	θ_max_ = 27.5°, θ_min_ = 2.4°
Absorption correction: multi-scan (*SADABS*; Bruker, 2001)	*h* = −8→8
*T*_min_ = 0.904, *T*_max_ = 0.988	*k* = −17→17
18931 measured reflections	*l* = −14→14

### Refinement

**Table:**

Refinement on *F*^2^	Primary atom site location: structure-invariant direct methods
Least-squares matrix: full	Secondary atom site location: difference Fourier map
*R*[*F*^2^ > 2σ(*F*^2^)] = 0.036	Hydrogen site location: inferred from neighbouring sites
*wR*(*F*^2^) = 0.105	H-atom parameters constrained
*S* = 1.05	*w* = 1/[σ^2^(*F*_o_^2^) + (0.0592*P*)^2^ + 0.4079*P*] where *P* = (*F*_o_^2^ + 2*F*_c_^2^)/3
2327 reflections	(Δ/σ)_max_ < 0.001
163 parameters	Δρ_max_ = 0.31 e Å^−^^3^
0 restraints	Δρ_min_ = −0.28 e Å^−^^3^

### Special details

**Table:**

Geometry. All e.s.d.'s (except the e.s.d. in the dihedral angle between two l.s. planes) are estimated using the full covariance matrix. The cell e.s.d.'s are taken into account individually in the estimation of e.s.d.'s in distances, angles and torsion angles; correlations between e.s.d.'s in cell parameters are only used when they are defined by crystal symmetry. An approximate (isotropic) treatment of cell e.s.d.'s is used for estimating e.s.d.'s involving l.s. planes.
Refinement. Refinement of *F*^2^ against ALL reflections. The weighted *R*-factor *wR* and goodness of fit *S* are based on *F*^2^, conventional *R*-factors *R* are based on *F*, with *F* set to zero for negative *F*^2^. The threshold expression of *F*^2^ > σ(*F*^2^) is used only for calculating *R*-factors(gt) *etc*. and is not relevant to the choice of reflections for refinement. *R*-factors based on *F*^2^ are statistically about twice as large as those based on *F*, and *R*– factors based on ALL data will be even larger.

### Fractional atomic coordinates and isotropic or equivalent isotropic displacement parameters (Å^2^)

**Table:**

	*x*	*y*	*z*	*U*_iso_*/*U*_eq_	
N1	0.27652 (15)	0.50895 (8)	0.56149 (9)	0.0150 (2)	
N2	0.15565 (15)	0.51345 (8)	0.31494 (9)	0.0165 (2)	
H2A	0.1890	0.5153	0.3913	0.020*	
O1	0.38783 (15)	0.30360 (6)	0.50820 (8)	0.0223 (2)	
O2	0.08964 (13)	0.29549 (6)	0.56245 (7)	0.0182 (2)	
O3	0.45903 (15)	0.26665 (6)	0.77965 (8)	0.0227 (2)	
O4	0.47246 (15)	0.35410 (6)	0.94390 (8)	0.0224 (2)	
H4A	0.5120	0.2999	0.9777	0.027*	
C1	0.31214 (17)	0.42864 (8)	0.63036 (10)	0.0135 (2)	
C2	0.38515 (17)	0.43413 (8)	0.75593 (10)	0.0137 (2)	
C3	0.41707 (18)	0.52530 (9)	0.80969 (11)	0.0155 (3)	
H3	0.4647	0.5310	0.8947	0.019*	
C4	0.37892 (18)	0.60781 (9)	0.73843 (11)	0.0166 (3)	
H4	0.3994	0.6707	0.7735	0.020*	
C5	0.31027 (18)	0.59644 (9)	0.61490 (11)	0.0161 (3)	
H5	0.2860	0.6528	0.5658	0.019*	
C6	0.26464 (18)	0.33299 (9)	0.56194 (10)	0.0151 (3)	
C7	0.44097 (18)	0.34292 (8)	0.82870 (10)	0.0148 (2)	
C8	0.13421 (18)	0.59568 (9)	0.25032 (11)	0.0177 (3)	
H8	0.1612	0.6565	0.2904	0.021*	
C9	0.07338 (19)	0.59273 (9)	0.12624 (11)	0.0170 (3)	
H9	0.0595	0.6512	0.0812	0.020*	
C10	0.03217 (17)	0.50302 (9)	0.06698 (10)	0.0148 (3)	
C11	0.05390 (19)	0.41949 (9)	0.13730 (11)	0.0180 (3)	
H11	0.0250	0.3576	0.1002	0.022*	
C12	0.11771 (19)	0.42708 (9)	0.26137 (11)	0.0187 (3)	
H12	0.1347	0.3699	0.3090	0.022*	

### Atomic displacement parameters (Å^2^)

**Table:**

	*U*^11^	*U*^22^	*U*^33^	*U*^12^	*U*^13^	*U*^23^
N1	0.0158 (5)	0.0161 (5)	0.0131 (5)	0.0009 (4)	0.0039 (4)	0.0014 (4)
N2	0.0177 (5)	0.0207 (5)	0.0106 (5)	−0.0012 (4)	0.0032 (4)	0.0010 (4)
O1	0.0308 (5)	0.0181 (5)	0.0215 (5)	0.0006 (4)	0.0130 (4)	−0.0027 (3)
O2	0.0197 (4)	0.0178 (4)	0.0156 (4)	−0.0025 (3)	0.0026 (3)	−0.0045 (3)
O3	0.0355 (5)	0.0131 (4)	0.0151 (4)	0.0017 (4)	0.0002 (4)	−0.0007 (3)
O4	0.0375 (5)	0.0165 (5)	0.0118 (4)	0.0072 (4)	0.0045 (4)	0.0034 (3)
C1	0.0128 (5)	0.0142 (5)	0.0136 (5)	0.0006 (4)	0.0038 (4)	0.0009 (4)
C2	0.0137 (5)	0.0143 (6)	0.0129 (5)	0.0005 (4)	0.0036 (4)	0.0002 (4)
C3	0.0163 (5)	0.0170 (6)	0.0128 (5)	0.0008 (4)	0.0035 (4)	−0.0006 (4)
C4	0.0183 (6)	0.0137 (6)	0.0174 (6)	0.0001 (4)	0.0045 (5)	−0.0016 (4)
C5	0.0164 (6)	0.0139 (6)	0.0180 (6)	0.0015 (4)	0.0048 (4)	0.0032 (4)
C6	0.0207 (6)	0.0143 (6)	0.0084 (5)	0.0023 (4)	0.0012 (4)	0.0020 (4)
C7	0.0147 (5)	0.0152 (6)	0.0130 (5)	−0.0013 (4)	0.0013 (4)	0.0000 (4)
C8	0.0200 (6)	0.0170 (6)	0.0163 (6)	−0.0001 (4)	0.0052 (5)	−0.0002 (4)
C9	0.0190 (6)	0.0165 (6)	0.0153 (6)	0.0009 (4)	0.0046 (4)	0.0027 (4)
C10	0.0120 (5)	0.0187 (6)	0.0135 (6)	−0.0009 (4)	0.0034 (4)	0.0016 (4)
C11	0.0218 (6)	0.0165 (6)	0.0146 (6)	−0.0036 (5)	0.0033 (5)	0.0001 (4)
C12	0.0213 (6)	0.0184 (6)	0.0156 (6)	−0.0028 (5)	0.0037 (5)	0.0035 (5)

### Geometric parameters (Å, °)

**Table:**

N1—C5	1.3446 (15)	C3—C4	1.3857 (16)
N1—C1	1.3457 (15)	C3—H3	0.9500
N2—C12	1.3330 (16)	C4—C5	1.3840 (17)
N2—C8	1.3432 (16)	C4—H4	0.9500
N2—H2A	0.8501	C5—H5	0.9500
O1—C6	1.2303 (15)	C8—C9	1.3807 (17)
O2—C6	1.2775 (15)	C8—H8	0.9500
O3—C7	1.2165 (15)	C9—C10	1.4030 (17)
O4—C7	1.3009 (14)	C9—H9	0.9500
O4—H4A	0.8501	C10—C11	1.3938 (17)
C1—C2	1.4009 (16)	C10—C10^i^	1.492 (2)
C1—C6	1.5245 (16)	C11—C12	1.3841 (17)
C2—C3	1.3915 (16)	C11—H11	0.9500
C2—C7	1.5007 (15)	C12—H12	0.9500
			
C5—N1—C1	119.02 (10)	O1—C6—C1	118.49 (11)
C12—N2—C8	121.10 (10)	O2—C6—C1	113.81 (10)
C12—N2—H2A	118.2	O3—C7—O4	124.97 (11)
C8—N2—H2A	120.7	O3—C7—C2	120.13 (10)
C7—O4—H4A	108.0	O4—C7—C2	114.88 (10)
N1—C1—C2	121.58 (10)	N2—C8—C9	120.66 (11)
N1—C1—C6	115.20 (10)	N2—C8—H8	119.7
C2—C1—C6	123.21 (10)	C9—C8—H8	119.7
C3—C2—C1	118.59 (10)	C8—C9—C10	119.71 (11)
C3—C2—C7	121.43 (10)	C8—C9—H9	120.1
C1—C2—C7	119.84 (10)	C10—C9—H9	120.1
C4—C3—C2	119.61 (11)	C11—C10—C9	117.85 (11)
C4—C3—H3	120.2	C11—C10—C10^i^	120.96 (13)
C2—C3—H3	120.2	C9—C10—C10^i^	121.19 (13)
C5—C4—C3	118.40 (11)	C12—C11—C10	119.75 (11)
C5—C4—H4	120.8	C12—C11—H11	120.1
C3—C4—H4	120.8	C10—C11—H11	120.1
N1—C5—C4	122.79 (11)	N2—C12—C11	120.93 (11)
N1—C5—H5	118.6	N2—C12—H12	119.5
C4—C5—H5	118.6	C11—C12—H12	119.5
O1—C6—O2	127.57 (11)		
			
C5—N1—C1—C2	−0.59 (17)	C2—C1—C6—O2	78.49 (14)
C5—N1—C1—C6	178.63 (10)	C3—C2—C7—O3	−162.59 (11)
N1—C1—C2—C3	1.26 (17)	C1—C2—C7—O3	13.12 (17)
C6—C1—C2—C3	−177.90 (10)	C3—C2—C7—O4	15.52 (16)
N1—C1—C2—C7	−174.57 (10)	C1—C2—C7—O4	−168.77 (11)
C6—C1—C2—C7	6.27 (17)	C12—N2—C8—C9	−0.43 (18)
C1—C2—C3—C4	−0.83 (17)	N2—C8—C9—C10	0.43 (18)
C7—C2—C3—C4	174.93 (11)	C8—C9—C10—C11	0.31 (18)
C2—C3—C4—C5	−0.21 (17)	C8—C9—C10—C10^i^	−179.48 (13)
C1—N1—C5—C4	−0.53 (18)	C9—C10—C11—C12	−1.05 (18)
C3—C4—C5—N1	0.93 (18)	C10^i^—C10—C11—C12	178.75 (13)
N1—C1—C6—O1	75.36 (14)	C8—N2—C12—C11	−0.34 (18)
C2—C1—C6—O1	−105.43 (13)	C10—C11—C12—N2	1.09 (19)
N1—C1—C6—O2	−100.72 (12)		

Symmetry codes: (i) −*x*, −*y*+1, −*z*.

### Hydrogen-bond geometry (Å, °)

**Table:**

*D*—H···*A*	*D*—H	H···*A*	*D*···*A*	*D*—H···*A*
N2—H2A···N1	0.85	1.90	2.7430 (14)	175
O4—H4A···O2^ii^	0.85	1.64	2.4782 (12)	171
C3—H3···O4^iii^	0.95	2.40	3.2055 (15)	143
C4—H4···O2^iv^	0.95	2.55	3.4324 (15)	155
C9—H9···O1^v^	0.95	2.41	3.3405 (15)	166
C11—H11···O1^vi^	0.95	2.52	3.4610 (15)	170
C12—H12···O3^vi^	0.95	2.19	2.9004 (15)	131

Symmetry codes: (ii) *x*+1/2, −*y*+1/2, *z*+1/2; (iii) −*x*+1, −*y*+1, −*z*+2; (iv) −*x*+1/2, *y*+1/2, −*z*+3/2; (v) −*x*+1/2, *y*+1/2, −*z*+1/2; (vi) *x*−1/2, −*y*+1/2, *z*−1/2.
